# Disrupted Structural Brain Network Organization Behind Depressive Symptoms in Major Depressive Disorder

**DOI:** 10.3389/fpsyt.2020.565890

**Published:** 2020-09-23

**Authors:** Jing Liu, Xiaopei Xu, Chunqing Zhu, Liyuan Luo, Qi Wang, Binbin Xiao, Bin Feng, Lingtao Hu, Lanying Liu

**Affiliations:** ^1^Department of Psychiatry, Tongde Hospital of Zhejiang Province, Hangzhou, China; ^2^Department of Radiology, The Second Affiliated Hospital of Zhejiang University School of Medicine, Hangzhou, China

**Keywords:** major depressive disorder, depressive symptoms, brain network, network analysis, diffusion tensor imaging

## Abstract

Major depressive disorder (MDD) is a severe and devastating condition. However, the anatomical basis behind the affective symptoms, cognitive symptoms, and somatic-vegetative symptoms of MDD is still unknown. To explore the mechanism behind the depressive symptoms in MDD, we used diffusion tensor imaging (DTI)–based structural brain connectivity analysis to investigate the network difference between MDD patients and healthy controls (CN), and to explore the association between network metrics and patients’ clinical symptoms. Twenty-six patients with MDD and 25 CN were included. A baseline 24-item Hamilton rating scale for depression (HAMD-24) score ≥ 21 and seven factors (anxiety/somatization, weight loss, cognitive disturbance, diurnal variation, retardation, sleep disturbance, hopelessness) scores were assessed. When compared with healthy subjects, significantly higher characteristic path length and clustering coefficient as well as significantly lower network efficiencies were observed in patients with MDD. Furthermore, MDD patients demonstrated significantly lower nodal degree and nodal efficiency in multiple brain regions including superior frontal gyrus (SFG), supplementary motor area (SMA), calcarine fissure, middle temporal gyrus (MTG), and inferior temporal gyrus (ITG). We also found that the characteristic path length of MDD patients was associated with weight loss. Moreover, significantly lower global efficiency of MDD patients was correlated with higher total HAMD score, anxiety somatization, and cognitive disturbance. The nodal degree in SFG was also found to be negatively associated with disease duration. In conclusion, our results demonstrated that MDD patients had impaired structural network features compared to CN, and disruption of optimal network architecture might be the mechanism behind the depressive symptoms and emotion disturbance in MDD patients.

## Introduction

Major depressive disorder (MDD), which affects more than 322 million people worldwide, has become the second leading contributor to chronic disease burden among all medical conditions (1) as measured “by years lived with disability”. MDD is characterized by at least one discrete depressive episode lasting for at least 2 weeks that is present across most situations (2). The discrete depressive episode includes clear changes in mood, interests and pleasure, changes in cognition and vegetative symptoms. Large-scale longitudinal studies indicated that MDD increases the risk of diabetes mellitus, heart disease, stroke, hypertension, obesity, cancer, cognitive impairment, and Alzheimer disease (3). Among all the studies trying to find the root of depressive symptoms of MDD, a significant amount of attention has been focused on the functional network changes in frontal-subcortical circuit (4, 5) and the role of network disruption in MDD has also been continuously explored using functional magnetic resonance imaging (fMRI) (6, 7). Now, MDD is regarded as a disorder of dysregulated neural networks, including the default mode network (DMN), the salience network (SN), the cognitive control network (CCN), the affective network (AN), and parts of the limbic system, rather than as a destruction of single brain regions (8). However, the results are inconsistent (9–11) regarding the network change of MDD, and the pathogenesis of MDD is still not clear. Given the heterogeneity of this disorder, identifying brain alterations is particularly important as this may indicate potential neural networks related to diagnose and treatment response, as one aspect of the biological markers of MDD. The exploration of biomarkers and timely and effective intervention for MDD is of great importance.

In addition to functional network, diffusion tensor imaging (DTI) has enabled the mapping of white matter (WM) pathways through which human brain is interconnected. Since DTI can be applied to visualize and measure WM pathways in the living brain (12), the relationship between structural abnormalities in specified brain regions and depressive symptoms in MDD (13, 14) were studied using this method. Past studies have suggested that WM abnormalities are associated with emotion disturbances (15, 16). Liao et al. (17) have demonstrated impaired WM integrity in individuals with MDD in studies using DTI, and aberrant connectivity between higher level cognitive structures and subcortical limbic structures was also found. Recently, brain connectivity analysis has been applied widely in understanding the interworking of brain function and this noninvasive tool has provided an unprecedented opportunity to further understand the basis of an individual’s vulnerability to MDD. Graph-based studies have shown topological disruption in anatomical networks of MDD, indicating a disconnection syndrome behind depressive symptoms (18). Several recent studies have revealed abnormal network interactions in patients with MDD (11, 19, 20). Yamada et al. (13) found that microstructural abnormalities in the anterior callosal fibers are associated with impairment of working memory and attention in MDD. However, so far there are only limited studies of structural brain network in MDD based on graph theoretical analysis.

The aim of present study was to examine the differences in structural brain network between MDD patients and healthy controls (CN) using graph theoretical analysis. We hypothesized that patients with MDD will demonstrate significantly lower global and regional network efficiency compared to those CN. Moreover, global and regional network metrics will be associated with the depression symptoms in those patients.

## Materials and Methods

### Participants

The MDD patients were recruited from outpatients and inpatients of Tongde Hospital of Zhejiang Province in Hangzhou, China. By the time of recruitment, all patients were experiencing a recurrent moderate or severe depressive episode according to the DSM-IV-TR (21) criteria, using the Structured Clinical Interview for DSM-IV (SCID-I/P, First MB, et al. New York State Psychiatric Institute, November 2002). All patients had a baseline 24-item Hamilton rating scale for depression [HAMD-24 (22)] score ≥ 21. All patients were right-handed and received no antidepressants or other psychotropic treatment within the 3 months prior to imaging experiment. Exclusion criteria of patients included: (1) first episode of depression; (2) pregnant or lactating women; (3) a history of brain injury surgery; (4) a history of cardiovascular, hepatic or renal diseases; (5) a history of manic, hypomanic, or mixed episodes; and (6) a history of receiving investigational drug treatment within the previous 6 months; and (7) a history of alcohol or drug abuse within the previous 12 months; Age, sex, and education matched CN were recruited from the community. The CN were screened for personal or family history of significant mental and physical illness. The study was approved by the Ethics Committee of Tongde Hospital of Zhejiang Province. All participants provided written informed consent before entering the study. Eventually, a total of 26 MDD patients (38.69 ± 12.54) and 25 CN (34.76 ± 6.25) were included.

### Functional Assessments

A baseline 24-item Hamilton rating scale for depression [HAMD-24 (22)] was investigated by two doctors who finished the required training for investigating. HAMD-24 score and seven factors scores, anxiety/somatization, weight loss, cognitive disturbance, diurnal variation, retardation, sleep disturbance, and hopelessness were assessed.

### Image Acquisition

Imaging acquisition was performed using a 3.0-Tesla scanner (Magnetom Trio Tim, Siemens) in the Department of Radiology at Tongde Hospital. A 16-channel birdcage head coil fitted with foam pads was used to fix each subject**’**s head and to limit head movement. Structural 3D high-resolution images were acquired at a voxel size of 1 mm^3^ isotropic using a T1-weighted gradient echo sequence, covering the entire brain and parallel to the anterior commissure–posterior commissure (AC–PC) line. The acquisition parameters are: flip angle = 9°; TR = 1,900 ms, TE = 2.48 ms, TI = 900 ms; slice thickness = 1.0 mm without gap; reconstruction resolution = 1 × 1 × 1 mm^3^; field of view = 512 × 512 mm^2^. Diffusion weighted images (DWIs) are consist of non-diffusion-weighted image (b0) and diffusion-weighted images along 30 gradient directions with a b-value of 1,000 s/mm^2^. DWIs were acquired using a single-shot, echoplanar sequence, and the parameters are: TR/TE = 8600/92 ms; number of transversal slices = 55; flip angle = 90°; NEX = 1; gradient directions = 30; field of view = 256 × 248 mm^2^; slice thickness = 2.0 mm without gap.

### Image Processing and Brain Network Construction

Detailed image processing steps for brain connectivity analysis are explained below.

#### Brain Parcellation

To parcellate the brain into cortical and subcortical brain regions, automated anatomical labeling (AAL) atlas was used on high resolution structural images, and the mask for each brain regions was transformed into each subject’s native space. Firstly, the subject’s structural images were registered to the corresponding DTI images with affine transformation. Then, non-linear transformation was used to register the native space structural images to the ICBM 152 template in the Montreal Neurological Institute’s (MNI) space. We then applied the inverse of the transformation matrix to the atlas, thereby transforming all brain ROIs into individual subject’s native space.

#### Diffusion MRI Tractography

For DTI preprocessing, using FMRIB’s Diffusion Toolbox, we registered all DWIs were first registered to the b0 image to correct for eddy current distortion and head motion. The diffusion tensor and its associated various diffusion parametric maps were obtained with the Diffusion Toolkit (http://trackvis.org/dtk/). To construct the WM fiber connections between the 90 brain regions, a deterministic streamline fiber tractography algorithm (the Fiber Assignment by Continuous Tracking algorithm, FACT) was used to obtain the DTI-based tractography using software implemented in the Diffusion Toolkit. A fractional anisotropy threshold = 0.2 and a fiber turning angle threshold = 45° were applied to the algorithm.

#### Brain Network

In order to describe the structural brain network configuration, a connectivity matrix needs to be constructed to characterize the structural pathways (i.e., network edges) between all brain regions. The UCLA Multimodal Connectivity Package was used to estimate connections amongst the 90 brain regions from the WM tractogram. In short, we count the number of WM fiber tracts originating from one region and ending in another region. The fiber count was quantified as the weight of each edge. After applying this method on all 90 brain regions, an undirected weighted network was constructed. To define the network edges and remove the spurious connections, a threshold value was selected for the fiber bundles (23–26), and a minimum threshold of fiber number (wij = 10, where wij is defined as the weight of the edge) between two regions was used. This threshold application reduced the risk of false positive rate due to not only noise but also limitations in the deterministic tractography; meanwhile, it guaranteed that the maximum connection size in the networks could be observed across all controls. The impact of different thresholds on the network configuration was also tested by setting threshold values of wij ranging from 5 to 15, and our results were not significantly influenced by this thresholding procedure. After the construction of structural network, the weighted matrix was converted into a binary matrix in which 1 was assigned to the edges with absolute fiber number greater than the threshold value (10 in our case), and 0 was otherwise assigned to edges with fiber number lower than the threshold value.

### Brain Connectivity Analysis

Graph theory was used to quantify the network efficiency and nodal characteristics of both weighted and binary brain network for all subjects. Each subject**’**s weighted network matrix was divided by the maximum entry to minimize the cross-subject differences between different subjects. Then, after normalizing the matrix, the Brain Connectivity Toolbox (27) was used to compute small-world properties, including clustering coefficient and characteristic shortest path length, as well as network efficiency, like global efficiency, and local efficiency. The characteristics of each region, such as the degree, clustering coefficient, betweenness centrality, and nodal efficiency, were also obtained.

To determine the nature of a structural network, we need to compare the characteristic shortest path length and clustering coefficient of the current network with a random network. For individual structural network, a set of 100 randomized networks was generated with the same number of edge number and degree distribution as the original network. A network was considered a small-world configuration if the normalized characteristic path length was close to one, the normalized clustering coefficient was much greater than one, and the small-worldness (σ = γ/λ) was greater than one (28).

### Statistical Analysis

Demographics were compared between CN and MDD patients using student t-test and Fisher’s exact test. Student t-tests were performed to evaluate the group difference between CN and MDD patients in all global and local network measures. To further test the robustness of group comparison analyses, we conducted the permutation tests to assess group differences in above-mentioned measures. For each permutation, each participant was randomly assigned to any of the two groups with the same size as the original MDD and CN groups. The mean differences between the two randomized groups were then computed. After repeating this permutation procedure 5,000 times, each distribution’s 95th percentile points were used as the critical values for a one-tailed test. The null hypothesis is that the probability of type I error is 0.05. R statistical software package (version 3.5, R Core Team, http://www.R-project.org/) and Exact Rank Tests Package were used for above-mentioned analyses. The association between all functional assessments and network measures were estimated using Spearman rank correlation after controlling for education. All analyses were adjusted for sex, age and duration of MDD. For those regional network metrics that tested positive for group comparisons, further correlation analyses were conducted between functional assessments and regional network measures in MDD group. For all the statistical analyses described above, we adopted Bonferroni correction for the problem of multiple comparisons, including correlation analysis and group comparisons. All the *p*-values reported were corrected. All statistical analyses were performed with SPSS 22.0 (SPSS, Chicago, IL) and a significance level of *p* < 0.05 was set for all statistical tests.

## Results

### Demographic and Clinical Characteristics

The demographics and clinical characteristics of the participants are shown in **Table 1**. There were no significant differences in age, gender, and education between CN and patients with MDD (see **Table 1**).

**Table 1:** Demographic and clinical characteristics of MDD patients and CN.

	MDD	CN	t/χ2	P
Sample size	26	25	–	–
Age (mean ± standard deviation)	38.69 ± 12.54	34.76 ± 6.25	t = 1.430	0.162
Gender (male/female)	7/19	10/15	χ2 = 0.981	0.244
Education level (high school or below/university or above)	8/18	4/21	χ2 = 1.545	0.324
Disease duration (months)	35.35 ± 41.32	–	–	–
Duration of untreated psychosis (months)	5.21 ± 5.39	–	–	–
HAMD total	27.65 ± 8.38	0.44 ± 0.92		
Anxiety/somatization	5.04 ± 2.34	0.12 ± 0.60		
Weight loss	0.46 ± 0.65	0		
Cognitive disturbance	4.46 ± 3.08	0.04 ± 0.20		
Diurnal variation	0.23 ± 0.43	0		
Retardation	7.88 ± 2.36	0.20 ± 0.41		
Sleep disturbance	4.19 ± 1.72	0.08 ± 0.28		
Hopelessness	5.38 ± 2.27	0		

MDD, Major depressive disorder; CN, Healthy controls; -, Not applicable.

### Global Network Alterations

**Figure 1:**
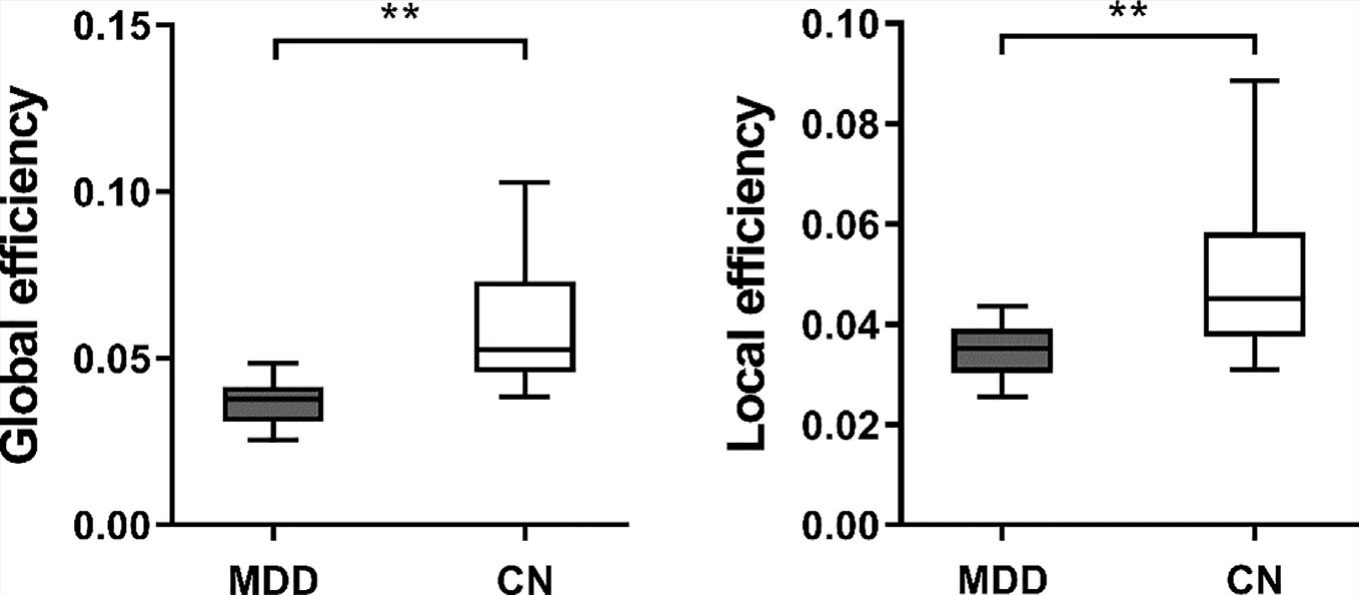
The boxplot of (mean ± standard deviation) of global and local efficiency of MDD patients and CN. MDD, Major depressive disorder; CN, Healthy controls. **At the 0.01 level significantly correlated (bilateral).

Both heathy control group and patient group had significantly larger clustering coefficient and relatively the same characteristic path length as compared to random network, which means they both demonstrated a typical small-world network. For weighted networks, when compared with CN, significantly higher characteristic path length (*p* < 0.001) and clustering coefficient (*p* < 0.001) were observed in patients with MDD. Furthermore, MDD patients also showed significantly decreased global (*p* < 0.001) and local efficiency (*p* < 0.001), as shown in **Figure 1**. For binary networks, the characteristic path length (*p* < 0.001) and clustering coefficient (*p* = 0.001) of MDD patients were significantly higher than those in CN, while both global (*p* < 0.001) and local efficiency (*p* < 0.001) of MDD patients were significantly decreased when compared to CN.

### Regional Network Alterations

**Figure 2:**
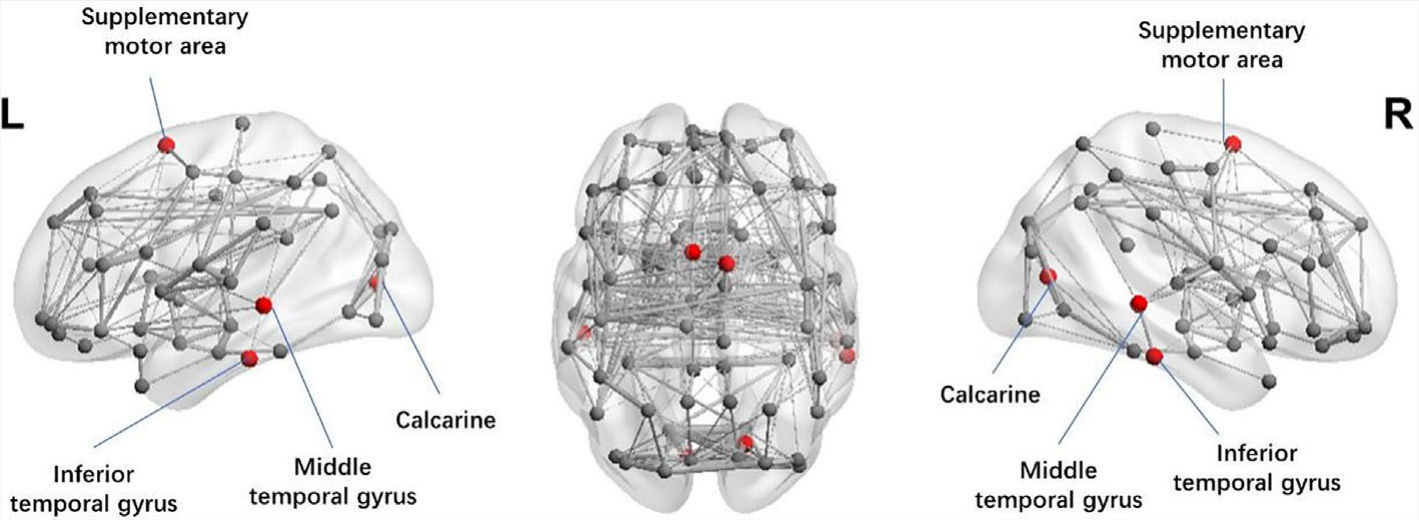
Illustration of the structural brain network of MDD patients showing brain regions with significant decreases in nodal efficiency (red spheres) as compared to CN. The width of network edge is weighted by the number of connections. MDD, Major depressive disorder; CN, Healthy controls.

We also investigated the regional network features in two cohorts. For weighted networks, significantly lower nodal degree in superior frontal gyrus (SFG) (*p* = 0.029), and lower nodal efficiency in supplementary motor area (SMA) (*p* = 0.036), calcarine fissure (*p* = 0.001), middle temporal gyrus (MTG) (*p* = 0.006), and inferior temporal gyrus (ITG) (*p* = 0.017) were observed in patients with MDD (see **Figure 2**). For binary networks, significantly decreased nodal degree in SFG (*p* = 0.001), and significantly decreased nodal efficiency in cuneus (*p* < 0.001) and middle frontal gyrus (MFG) (*p* < 0.001) were found in MDD when compared to CN.

### Associations Between Network Measures and Functional Assessments

Most importantly, when we studied the relationship between weighted network metrics and clinical variables (see **Table 2**), we found that the characteristic path length in MDD patients was associated (*p* = 0.031) with weight loss. Also, the global efficiency was negatively correlated with total HAMD score (*p* = 0.027), anxiety somatization (*p* = 0.015) and cognitive disturbance (*p* = 0.037) in patients with MDD. For binary network metrics, only the local efficiency (*r* = −0.417, *p* = 0.048) was negatively associated with cognitive disturbance in MDD patients. We also found that the nodal degree of MTG was negatively correlated with the cognitive disturbance (*r* = −0.469, *p* = 0.016) when we examined the relationship between regional network features and clinical variables. The nodal degree in SFG was also found to be negatively associated with disease duration (*r* = −0.424, *p* = 0.031). However, no association was found between diurnal variation, retardation, sleep disturbance, hopelessness and network metrics.

**Table 2:** Associations between weighted network measures and functional assessments.

		Clustering coefficient	Characteristic path length	Global efficiency	Local efficiency
HAMD total	r	0.069	0.060	−0.518*	0.038
	p	0.755	0.785	0.027	0.864
Anxiety/somatization	r	0.217	0.115	−0.469**	0.143
	p	0.320	0.600	0.015	0.515
Weight loss	r	−0.120	0.463*	0.114	0.086
	p	0.584	0.031	0.604	0.695
Cognitive disturbance	r	−0.222	-0.104	−0.417*	−0.145
	p	0.309	0.636	0.037	0.510
Diurnal variation	r	−0.149	0.252	−0.107	0.066
	p	0.498	0.245	0.626	0.764
Retardation	r	0.045	0.115	0.232	0.134
	p	0.837	0.602	0.287	0.543
Sleep disturbance	r	0.389	0.123	−0.386	0.044
	p	0.067	0.576	0.069	0.841
Hopelessness	r	0.047	−0.115	−0.113	−0.012
	p	0.830	0.601	0.608	0.957

*At the 0.05 level significantly correlated (bilateral).

**At the 0.01 level significantly correlated (bilateral).

## Discussion

Recently, there has been greater focus on the mechanism behind the emotion disturbance in MDD patients. Studies on the emotion of MDD have centered on abnormal anatomical and functional connectivity (29, 30). From the perspective of network topological architectures, imbalanced functional integration has been increasingly reported in MDD (31, 32), and we are among the firsts to use graph theoretical analysis to explore the mood disturbance and cognitive function in MDD in this study. Overall, graph theoretical analysis is a mathematical framework describes the relationship between regions with nodes and edges representing brain regions and fiber track connections between regions respectively. It enables studies in brain anatomical networks and network topology analysis, hence provides modern researchers a new opportunity to investigate the complex organization of brain from a systematic view. Brain connectivity analyses have repeatedly demonstrated that the human brain is a small world network, and small-worldness was used to measure the balance between integration and segregation of a specific network (33). In current study, we examined the network features of MDD patients and CN. Compared with healthy subjects, patients with MDD showed significantly higher characteristic path length and clustering coefficient. Significantly decreased global and local efficiency in MDD were also observed. Clustering coefficient of a network quantifies to what extent the neighboring regions are connected with each other, thus is considered as the ability of a network to process localized information (34). With an increased clustering coefficient, connections between functional related or anatomical neighbor regions were densely connected, and those processes benefit greatly from efficient clustered connections between topological neighbors might be affected (35). Considering the balance of segregation and integration of brain network plays an important role in supporting the cognitive function of the brain (36, 37). Our findings suggested that the segregation information processing ability of MDD patients was impaired, which is in accordance with the impaired cognitive function in MDD patients. Meng et al. (38) found increased path lengths and decreased global efficiency in MDD patients, which is in accordance with our findings. However, Zhang et al. (9) observed lower characteristic path lengths and higher global efficiency in first-episode drug-naive depressive patients, which is not in line with our findings. Our study included patients with recurrent MDD may explain the differences. Another study investigating the whole-brain functional networks in MDD found significantly increased local efficiency (11), which is not in line with our findings. The fact that Ye et al. (11) included severe depression while patients in the present study were experiencing a recurrent moderate or severe depressive episode may account for the inconsistent results. Global efficiency quantifies the communication efficiency between long-range connections that facilitate functional integration. On the other hand, local efficiency is an indicator of regional network’s fault tolerance, which reflects functional isolation (39). With decreased efficiency in both global and local network, which is in line with our hypothesis, the communication between remote and nearby brain regions would be largely compromised. Those results indicated that the balance between integrated and segregated information processing ability was disrupted, and those processes related functions were impaired in patients with MDD. This might explain the depressive symptoms often observed in those patients.

According to our regional analysis, SFG, SMA, calcarine fissure, MTG, and ITG had reduced regional connections in patients with MDD. This suggested that those regions might be of great significance in the etiology of depression and might need more attention during future clinical management. SFG is part of the DMN and is related to emotional disorder and higher cognitive function (40, 41). Past study using a voxel-wise, data-driven, graph-based approach showed significantly reduced regional connections in SFG of MDD patients (30), which is in accordance with our findings. Du et al. (42) have confirmed the involvement of the SFG in the neural network dedicated to working memory. Moreover, decreased functional connectivity in regions including the superior temporal gyrus (STG), the orbital frontal cortex (OFC), and calcarine has been found in patients with MDD (43–45), which may explain the lower efficiency we found in this study. The SMA is associated with motor execution and vigilance performance (46), while both MTG and ITG are related to two-dimensional and three-dimensional cognitive learning. To further elaborate, MTG is associated with different processes, such as considering distance, identifying known faces, and accessing word meanings while reading (47), while ITG may be involved in face perception (48), and in the recognition of numbers (49). Thus, our results suggested that the ability of information processing is compromised in patients with MDD. By using fMRI, Kalin et al. (50) found that increased brain activation was associated with severer depression symptoms. Studies have found that patients with MDD had gray matter reduction in temporal lobe (51–53). The neural circuits through which connecting the temporal and parietal lobes play an important role in emotional regulation (54). More importantly, with further evidence we found in the association between network metrics and clinical variables, we are confident to say that disrupted global and regional network configuration might be the mechanism behind depressive symptoms in MDD patients.

Most importantly, we found that the global efficiency was negatively correlated with total HAMD score, anxiety/somatization and cognitive disturbance in patients with MDD. Researchers usually assess depressive severity *via* HAMD and the total HAMD score reflects the overall severity of depression. As described in the above sections, global efficiency is attributed to integrate information. Our results suggest that decreased network integration is associated with more severe depressive symptoms. Our finding was in accordance with a past study which showed that normalized global efficiency was negatively correlated with depression severity in patients with MDD (55). Anxiety symptoms of MDD in this study were defined using a 24-item Hamilton depression rating scale (22) anxiety/somatization factor. The anxiety/somatization factor consists of anxiety (psychic), anxiety (somatic), somatic symptoms (gastrointestinal), somatic symptoms (general), hypochondriasis, and insight (56). It is estimated that 40% to 60% of patients with MDD have anxiety symptoms (57) and anxiety has been identified as one of the core features of MDD (58). Anxiety symptoms can have a vital impact on the course of a depressive illness, with delayed recovery, increased risk of relapse, greater disability and increased suicide attempts (59). MDD patients with high anxiety levels are more prone to severe depression and functional impairment (60). Somatization refers to a trend of experiencing and communicating psychological distress and seeking medical help in the form of somatic symptoms (61, 62). Somatoform disorders, including somatization disorder, are prevalent psychiatric disorders that are highly comorbid with depression (63, 64). Cognitive theories suggest that cognitive biases affect the etiology and maintenance of somatoform disorders (65). Thus, we speculated that the lower network integration might be attributed to deterioration in the anxiety/somatization of MDD patients in current study, and decreased network integration might have a negative effect on cognitive function. However, compared with nonanxious depression, Delaparte et al. (66) found no significant differences of fractional anisotropy (FA) in the amygdala and uncinate fasciculus in anxious depression and no significant associations between measures of anxiety and depression, and structural connectivity measures. The diagnostic criteria and different methodology of the study might contribute to the conflicting results of the impact of anxiety or depression. The cognitive disturbance factor consists of self-guilt, suicide, agitation, depersonalization/decreolization, paranoid symptoms, and obsessive-compulsive symptoms (56). Cognitive dysfunction has also been proved to be worsened with increasing severity of depressive symptoms in currently employed individuals (67). The negative relation we found between cognitive disturbance and global efficiency may indicate that decreased network integration is correlated with more severe cognitive disturbance. Moreover, we found that MDD patients with longer characteristic path length had more weight loss. Weight loss is common among patients with MDD clinically. To examine the impact of weight loss on cognition in patients with MDD and bipolar disorder, Restivo et al. (68) reported that obesity may have a negative effect on cognition that is exacerbated in the presence of a mood disorder. Our finding indicates that MDD patients with higher characteristic path length are experiencing more weight loss. This might explain the changes in eating behaviors, appetite, and weight dysregulation in MDD. In addition, we found that the nodal degree in the MTG was negatively correlated with cognitive disturbance. Nodal degree is defined as the number of connections a node has to the rest of the network, and it is considered to be a measure of how a node interacts structurally or functionally with the network (69). The region that has a higher degree represents more functional connections with other brain regions in the network. We speculated that decreased nodal degree of the MTG in MDD patients might reflect more severe cognitive dysfunction. With the development of MDD, these regions might be damaged and lead to cognitive disturbance. What’s more, we found that the nodal degree in SFG was negatively correlated with disease duration, indicating the longer the illness duration, the more impairment of the SFG. Disease duration has been reported to be associated with WM disruption in MDD (9, 70). Our finding revealed the association between cognitive dysfunction and disease duration as mentioned above. These results verified the existence of WM structural network disruption and the association between structural network properties and depressive state.

Several limitations of the present study should be addressed. First, the sample was relatively small; therefore, a larger sample is needed to further confirm the results. Second, we applied network analysis in our study, which analyzed one dimensional feature independently. In a future study, multivariable-based predictive analysis with support vector machines could provide clearer information about changes in brain function in MDD with larger samples.

## Conclusion

Our results show that MDD had a significant increase in characteristic path length as well as clustering coefficient and decrease in global and local efficiency. Lower degree in SFG and lower nodal efficiency in regions like SMA, calcarine fissure, MTG, and ITG were also observed. In addition, we found that network metrics were associated with depression related functional assessments including weight loss, total HAMD score, anxiety somatization, and cognitive disturbance in patients with MDD. The nodal degree in SFG was also found to be negatively correlated with disease duration. The findings might give insight to the mechanism behind the mood disturbance in MDD and provide potential biomarkers for clinic treatment of MDD.

## Data Availability Statement

The datasets presented in this article are not readily available because: The datasets are the property of Tongde Hospital of Zhejiang Province, are only available upon reasonable request. Requests to access the datasets should be directed to LL, balindaliu@163.com.

## Ethics Statement

The studies involving human participants were reviewed and approved by Tongde Hospital of Zhejiang Province. The patients/participants provided their written informed consent to participate in this study.

## Author Contributions

LLi and BF conceived the study. CZ, LLu, QW, BX, and LH collected the data. XX analyzed the data. JL and XX drafted the paper.

## Funding

This research was supported by the National Natural Science Foundation of China (Grant No. 81403502), Zhejiang Provincial Major Research Projects of Traditional Chinese Medicine (Grant No. 2018ZY002), and Zhejiang provincial science and technology program (Grant No. 2018F10033).

## Conflict of Interest

The authors declare that the research was conducted in the absence of any commercial or financial relationships that could be construed as a potential conflict of interest.
